# Structural Complexity, Network Formation and Spontaneous Chirality in Soft-Matter Self-Assembly

**DOI:** 10.1021/acs.accounts.6c00103

**Published:** 2026-05-11

**Authors:** Yu Cao, Carsten Tschierske, Feng Liu

**Affiliations:** † Shaanxi International Research Center for Soft Matter, State Key Laboratory for Mechanical Behavior of Materials, Xi’an Jiaotong University, Xi’an, 710049, P. R. China; ‡ Institute of New Concept Sensors and Molecular Materials, Shaanxi Provincial Key Laboratory of New Concept Sensors and Molecular Materials, Xi’an Jiaotong University, Xi’an, 710049, P. R. China; § Institute of Chemistry, Martin Luther University Halle-Wittenberg, Kurt-Mothes-Straße 2, 06120, Halle, Germany

## Abstract

In modern science, chemists excel at programming interactions and functionalities on the molecular scale to design and create novel materials with diverse and tailored functionalities. Self-assembly, a major mechanism that amplifies these interactions, enables the emergence of well-defined structures at higher levels and larger length scales. The intricate interplay among molecular-scale properties (polarity, chirality, etc.), specific intermolecular interactions (hydrogen bonding, dispersion forces, π-stacking, etc.), nanoscale segregation, and global order gives rise to complex self-assembly behaviors that hold great promise for generating materials with novel properties and functions. While self-assembly has been extensively studied in solutions, on surfaces, and within solid-state materials, the understanding of complex soft self-assembly in highly dynamic but ordered fluids still remains in its infancy due to the interplay between entropic and enthalpic contributions. In this Account, we elucidate complex soft self-assembly in 3D networks formed by simple organic molecules involving π-conjugated rod-like building blocks. Two different types of compounds derived from linear π-conjugated polyaromatic rods have been designed, and their soft self-assembly was studied by different methods including synchrotron X-ray scattering and resonant soft X-ray scattering. One type of compound, called polycatenars, has alkyl chains attached to both ends, and the other one, the bolapolyphiles, have linear or branched alkyl chains side-on attached and polar glycerols at each end. Both types of compounds form network phases, differing in the orientation of the rods, with respect to the struts forming the network. In the first group, the rods are organized perpendicular (transversal) to the network direction which allows them to develop an intermolecular helical twist. These supramolecular helices propagate chirality through space and induce mirror-symmetry breaking in soft and fluid systems, which is highly relevant to the spontaneous emergence of uniform chirality, especially biological chirality. In the bolapolyphiles, the polar glycerols organize into supramolecular spheres, which are interlinked by bundles of parallel arranged rods forming the struts interconnecting the spheres into networks, in this case with the rods aligned parallel (longitudinal) to the network. These bundles of parallel rods can be considered as bonds, having defined lengths, linking the supramolecular spheres at the junctions with coordinate numbers ranging from 3 to 14. In total, 8 different networks, divided into single-, double-, and triple-networks, have been found, among them those formed by cubic, octahedral, and tetrahedral frames, including the I-WP network and the A15 type Frank Kasper network. Here, structural complexity arises from the delicate balance between optimizing sphere-packing and minimizing infinite periodic minimal surfaces. In summary, by leveraging specifically designed low molecular weight amphiphilic or polyphilic rod-like molecules equipped with multiple interactions, we have successfully extended the frontiers of programmable self-assembly from the domain of solid-state materials into the realm of soft matter, liquid crystals, and isotropic liquids. The insights into structural complexity and symmetry breaking have profound implications for understanding the emergence of chirality, as well as for the rational design of advanced soft materials. Potential applications include soft addressable and multifunctional structural, optical, chiroptical, and electronic materials.

## Graphical abstract

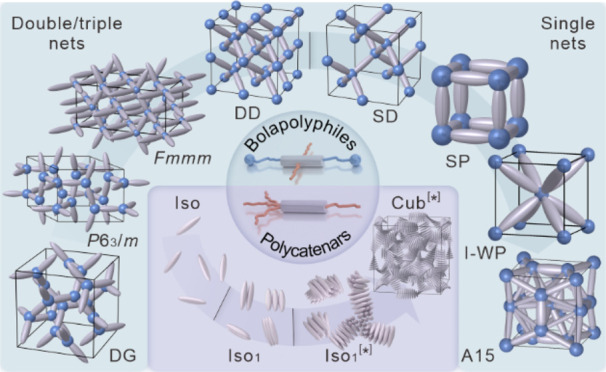

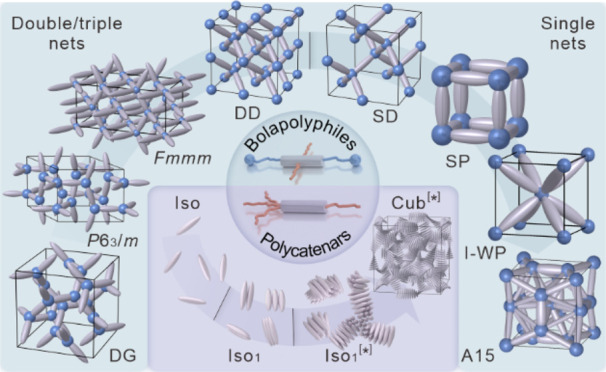

## KEY REFERENCES

-

Dressel, C. ; Reppe, T. ; Prehm, M. ; Brautzsch, M. ; Tschierske, C. Chiral self-sorting and amplification in isotropic liquids of achiral molecules. Nat. Chem. 2014, 6, 971–977.25343601 10.1038/nchem.2039 *The first example of spontaneous chirality generation and amplification in isotropic liquids is reported, which sheds light on homochirality generation.* ^1^
-

Cao, Y. ; Alaasar, M. ; Zhang, L. ; Zhu, C. ; Tschierske, C. ; Liu, F. Supramolecular meso-trick: ambidextrous mirror symmetry breaking in a liquid crystalline network with tetragonal symmetry. J. Am. Chem. Soc. 2022, 144, 6936–6945.35394276 10.1021/jacs.2c01511 *The competition between enantiophobic and enantiophilic self-assembly of distinct hierarchies and its influence on the spontaneous supramolecular chirality generation of achiral polyphiles is elucidated*.^2^
-

Chen, C. ; Poppe, M. ; Poppe, S. ; Wagner, M. ; Tschierske, C. ; Liu, F. Tetrahedral liquid crystalline networks: an A15-like Frank-Kasper phase based on rod-packing. Angew. Chem. Int. Ed. 2022, 61, No. e202203447.10.1002/anie.202203447PMC932182135470526 *The first Frank-Kasper A15 network phase composed by unicontinuous networks is deciphered to follow Delaunay Triangulation, which represents a new mode of F–K phase as the topological dual of conventional micellar type following Voronoi Cell.* ^3^
-

Cai, X. ; Hauche, S. ; Poppe, S. ; Cao, Y. ; Zhang, L. ; Huang, C. ; Tschierske, C. ; Liu, F. Network phases with multiple-junction geometries at the gyroid–diamond transition. J. Am. Chem. Soc. 2023, 145, 1000–1010.36603102 10.1021/jacs.2c10462 *A novel routine of double gyroid to double diamond network transition is discovered, which is controlled by the competition between minimizing minimal surface and densest spherical packing of junctions.* ^4^

## 1 Introduction

Nature has produced the most intricate and complex chemical systems by soft self-assembly of molecules into larger structures, guided by weak intermolecular interactions.^5^ While self-assembly through coordinative and noncovalent bonding has been extensively studied in solutions, on surfaces, and within solid-state materials (Figure 1),^6–13^ the understanding of *soft self-assembly in highly dynamic, ordered fluids* still remains in its infancy due to the complex interplay between entropic and enthalpic contributions. This soft mode of self-assembly is particularly important for the understanding of the emergence of complexity and functionality in ordered systems, ranging from liquid crystalline (LC) nanostructures^14–16^ to the sophisticated functional structures in biosystems.^17–21^ Our focus is on the directed molecular design and structural elucidation of new soft self-assembly modes by using small minimalistic model compounds. Herein, we will summarize our recent work on soft network structures formed by the self-assembly of π-conjugated rodlike building blocks in the liquid and LC state. Two different types of functionalized π-conjugated rods are considered (Figure 2).

The first type, the so-called polycatenar compounds, has a number of >2 flexible alkyl chains distributed to both ends of a π-conjugated rodlike unit (Figure 2a).^25,26^ Besides minimizing excluded volumes,^27,28^ the major driving forces of polycatenar ordering are nanosegregation,^29^ arene–arene interaction,^30^ and dispersion forces (Figure 2b).^31^ Typically, the π-conjugated rods form molecular rafts that then self-assemble into larger structures, controlled by the design of core structure and end-chains (Figure 2c). Our focus is on the formation of helical supramolecular networks that propagate chirality through space and induce *mirror-symmetry breaking* in soft and fluid systems.

The second type combines the rodlike cores with mutually incompatible groups at the ends and the sides, leading to polyphiles.^14,32^ Focus is on the bolapolyphiles composed of a π-conjugated rod with long aliphatic side-chains and glycerol end groups (Figure 2d) being capable of forming cooperative and dynamic hydrogen bonding.^33^ In previous work this concept was used to produce a large number of LC honeycombs, representing tilings in 2D crystallographic plane, including pentagon tilings, dual tessellations, multicolor tilings and quasiperiodic structures.^14,34–36^ The general feature of these honeycombs is that the rodlike units align parallel in ribbons and the hydrogen bonding between the sticky end groups fuse them to honeycombs, their interior being filled by the side-chains (Figure 2f, left). The side-chain volume with respect to the core length determines the shape of the polygonal cells, being additionally affected by steric and geometric frustration (Figure 2e).^32,37,38^ Herein, we focus on new LC network structures formed by side-chain expansion beyond the honeycombs (Figure 2f, right). The general focus of our work is on the various types of soft networks and an understanding of the fundamental concepts of the design of soft matter with enhanced structural complexity.

## 2 ENGINEERING HELICAL SELF-ASSEMBLY IN SOFT MATTER

### 2.1 Symmetry Breaking by Chirality Synchronization in Liquids

The surprising observation of spontaneous segregation of an isotropic liquid of *achiral* molecules into a fluid conglomerate of optically active domains (Iso_1_^[*]^) was first made by polarized optical microscopy (POM)^1^ of compound **1** (Figure 3a). Upon cooling, the highly fluid and isotropic liquid of **1** with distinct bright and dark domains of equal total area could be observed by polarizing microscopy in depolarized mode, i.e., by rotating the analyzer clockwise away from the 90° position by a small angle. The bright and dark domains can be reversed if the analyzer is rotated anticlockwise, indicating ambidextrous mirror symmetry breaking in the liquid state (Figure 3b).^2,39–41^ A typical feature is a broad hump in the differential scanning calorimetry (DSC) temperature scans in the achiral isotropic liquid, just before the transition to Iso_1_^[*]^ (Figure 3e),^41,42^ which is also found in compound **2** (Figure 3d). In this temperature range, a continuous increase of intensity and decrease of peak width of the diffuse small-angle X-ray scattering (SAXS) (Figure 3f) indicates a growing coherence length of rod packing in rafts (clusters). This is interpreted as a continuous transition from an almost molecular liquid (Iso) via an isolated cluster liquid to a liquid with strongly interacting clusters (Iso_1_) (Figure 3g). After crossing a certain coherence length of aggregation a reversible transition to the chiral domain texture of the Iso_1_^[*]^ phase takes place by spinodal unmixing associated with a small, but sharp DSC peak (Figure 3e).^1^ This indicates that enantiophobic chirality synchronization^43^ in this fluid is associated with a (first-order) phase transition.^41,42^ Polycatenars with transient helical conformations (Figure 3c) and the same twist sense pack more closely in helical aggregates, which creates a bias for the transient helical molecular conformations. As the helical aggregates grow, there is an increasing transmission of the helicity sense, and after crossing a certain critical correlation length of helix sense synchronization, macroscopic symmetry breaking takes place. In this way, supramolecular chirality is generated and amplified from molecular conformational chirality via cooperative helical packing in local aggregates which then further interact via local network formation and lateral interhelical interactions in Iso_1_^[*]^. Thus, the entropic penalty of unmixing of enantiomorphic conformers is more than compensated by the enthalpic gain due to denser packing after chirality synchronization.^43^ Due to the involved cooperativity a relatively small energetic difference between transiently chiral conformers (Figure 3c) is sufficient to achieve mirror symmetry breaking at temperatures even above 200 °C.^41,42,44^ This is supported by recent work with related polycatenars^45^ and by simulations using different models.^46–49^

**Figure 1:**
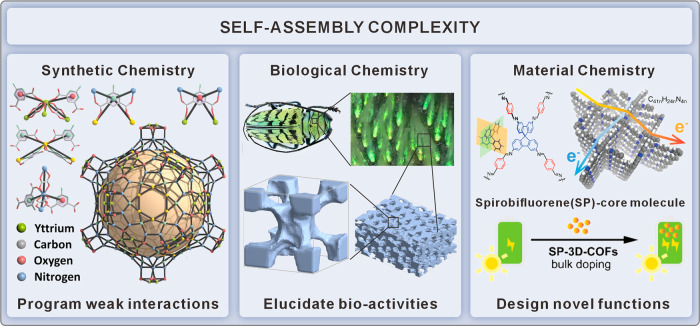
Structural complexity as the cutting-edge topic in various aspects of chemistry.^22–24^ [Reproduced with permission from ref 22, Copyright 2021, Royal Society of Chemistry; from ref 23, Copyright 2024, Elsevier; and from ref 24, Copyright 2024, American Chemical Society.]

**Figure 2:**
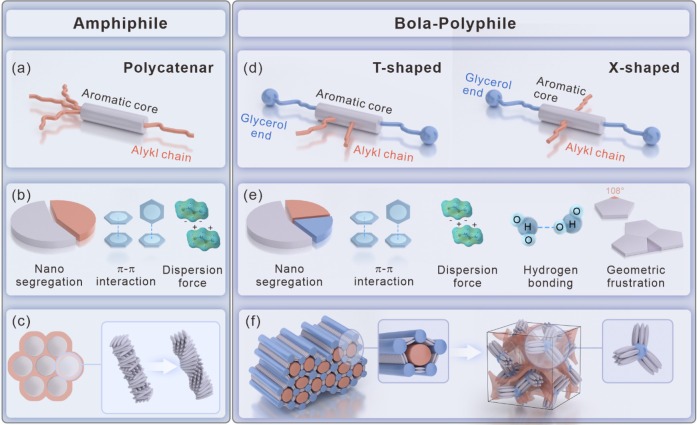
(a) Polycatenar amphiphiles and (d) bolapolyphiles discussed in this account, (b, e) their intermolecular interactions, and (c, f) dominating modes of soft self-assembly.

In these ambidextrous mirror symmetry broken liquids, homochirality can be achieved by traces of homochiral dopants at concentrations as low as 10^–3^–10^–8^ mol %, indicating a strong “chirality amplification power”.^1^ Uniform chirality can also be achieved by a transition to a chiral cubic phase on cooling (Cub^[*]^/*I*23) if seed formation is slow compared to the growth of Cub^[*]^/*I*23 from Iso_1_^[*]^. Then, a homochiral seed first formed in one of the enantiomeric domains can grow throughout the whole sample and reverses the helix sense in the fluid domains having opposite helicity, leading to complete mirror symmetry breaking.^1,50^ This chirality synchronization process provides a possible route to spontaneous macroscopic homochirality generation in the liquid state, as required for the development of uniform biochirality.^50,51^

### 2.2 Enantiophilic and Enantiophobic Chirality Synchronization in Soft Helical Networks

Upon further cooling, most symmetry broken liquids transform into soft helical networks with cubic symmetry, being either chiral or achiral.^44^ Often an achiral double gyroid (DG) cubic phase, whose space group is *Ia*3̅*d*, is formed (Figure 4a–d). The structure is composed of two interwoven networks with opposite network chirality, separated by Schoen’s G minimal surface, thus representing an achiral *meso*-structure, i.e., an initially formed chiral Iso_1_^[*]^ phase becomes achiral at the transition to *Ia*3̅*d*.^44^ By applying resonant soft X-ray scattering (RSoXS) to the DG phase of compound **3** (Figure 4a,b), we successfully revealed the molecular packing and supramolecular chirality generation mechanism in the LC network.^52^ RSoXS is a scattering technique that works at the absorption edges of certain elements. Especially useful is the scattering at the K-edge of C, as it can be applied to most organic compounds. With a linearly polarized beam, RSoXS identifies the bond orientation, resulting in signals forbidden or weakened by conventional SAXS, which contain information on helical molecular packing. Thus, RSoXS is of high potential for recognizing chirality, particularly in the field of organic materials.^53^

**Figure 3:**
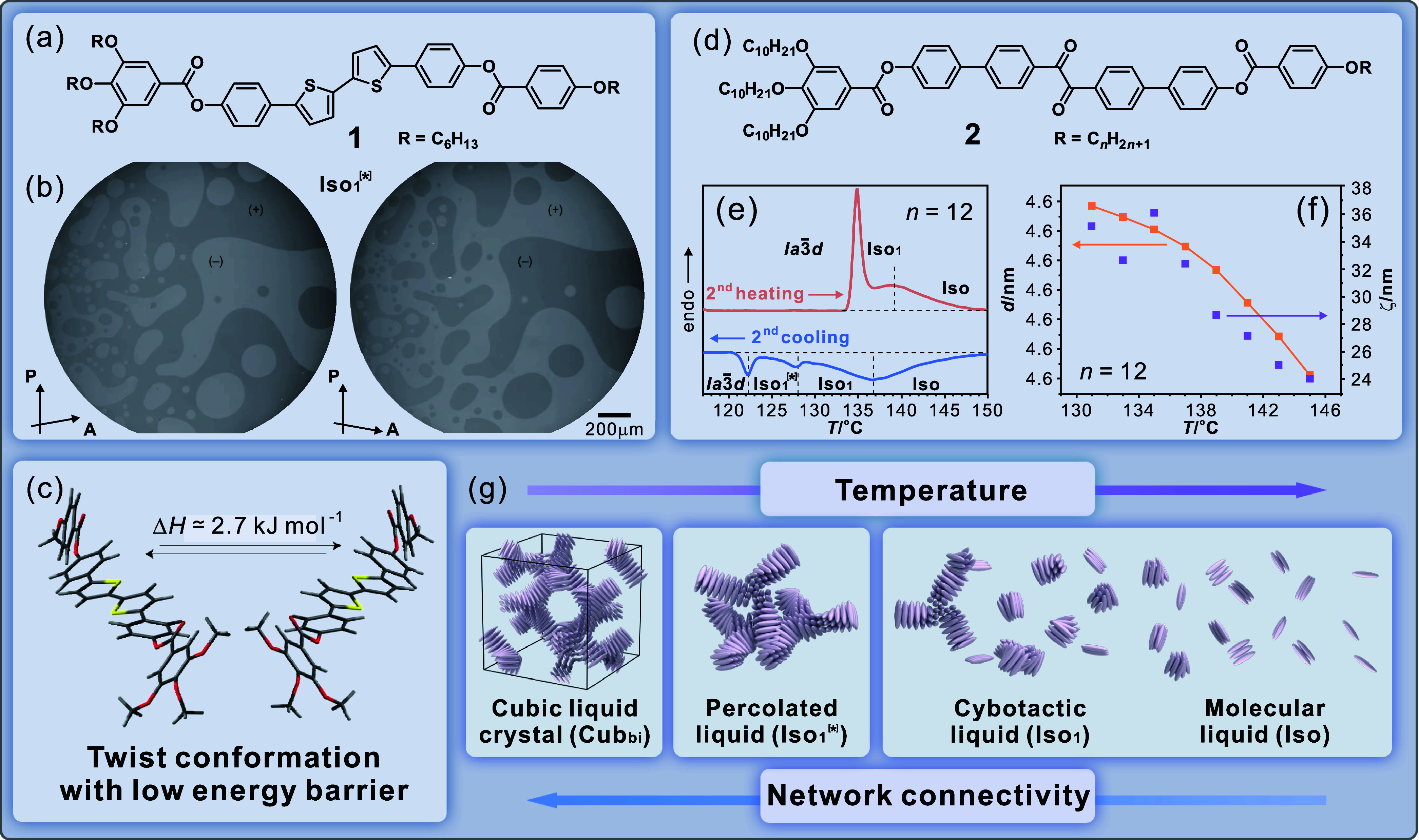
(a) The first achiral polycatenar compound **1** forming an isotropic liquid conglomerate (Iso_1_^[*]^). (b) Depolarized POM textures of Iso_1_^[*]^ phase confirming areas of opposite chirality. (c) The DFT calculation suggests helical energy minimum conformers and low enantiomerization energy of the twisted aromatic backbone.^1^ (d) Compound **2** exhibiting the same Iso_1_^[*]^ phase with (e) typical DSC trace with huge hump during the Iso–Iso_1_ transition and (f) growth of the coherence length of the clusters in the Iso–Iso_1_ range derived from temperature-dependent SAXS. (g) Schematic model showing the transition from a molecular (Iso) via a cybotactic (Iso_1_) to a mirror symmetry broken network liquid (Iso_1_^[*]^) before long-range order develops in the cubic network LC.^41^ [Reproduced with permission from ref 1, Copyright 2014, Springer Nature, and ref 41, Copyright 2020, Royal Society of Chemistry.]

Taking compound **3** as an example (Figure 4a), three bicontinuous network phases can be observed upon cooling.^2^ It first self-assembles into a DG phase at the highest temperature. With SAXS the *Ia*3̅*d* space group was confirmed and the electron density (ED) map of the DG phase was reconstructed (Figure 4c). RSoXS exhibits two resonant signals that are strongly dependent on the incident beam energy. The two signals can be indexed as (110) and (200), both are forbidden by the extinction rules of *Ia*3̅*d* space group (Figure 4b). With resonant signal simulation, we successfully elucidated that the aromatic backbones of compound **3** form supramolecular helices along the DG network, meeting with each other at the centers of three-way junctions without any discontinuity (Figure 4c, d).^52^ The sense of supramolecular helices follows the chirality of the two interwoven networks, becoming opposite of each other. Thus, there are three levels of helical twist determining the phase chirality, the helicity of molecular conformations (molecular helicity, Figure 3c), the helicity of the organization of the molecules within these rafts and networks (supramolecular helicity, Figure 4a), and the enantiomorphic shape of the two networks forming the DG (net helicity, Figure 4c).

Upon cooling, another bicontinuous network—in this case, a tetragonal phase (Tet_bi_)—appears as an intermediate phase^2^ (Figure 4e). The reconstructed ED map suggests that Tet_bi_ is a deformed DG phase by stretching the cubic lattice along the *c*-direction. The networks, retaining the three-way junctions, are still interwoven with each other as in DG, but having chiral space groups *P*4_1_2_1_2/*P*4_3_2_1_2, which is verified by the extinction rules of both SAXS and RSoXS, indicating that the phase is chiral. With help of RSoXS and resonant signal simulation, we unveil the molecular packing inside the tetragonal lattice. The molecular helix along one network unwinds into an overall achiral random packing, while the helix sense along the other enantiomorphic network is retained. The partial network racemization breaks the mirror symmetry of the interwoven networks, generating phase chirality (Figure 4f, g).^2^

At the lowest temperature, the symmetry changes at the transition to another cubic phase with much larger lattice parameter compared to *Ia*3̅*d.* At first, this kind of cubic phase was indexed to an *Im*3̅*m* lattice,^54^ but the discovery of mirror symmetry breaking in combination with electron density reconstructions lead to a structure composed of three interwoven *achiral* networks with three-way junctions.^44,55^ In this cubic phase with three achiral nets, i.e., without influence of net helicity (Figure 4h, i), the supramolecular helices can assume a synchronized uniform helix sense, leading to a macroscopic chiral cubic phase with the *I*23 space group (Figure 4i, j).^56,57^

**Figure 4:**
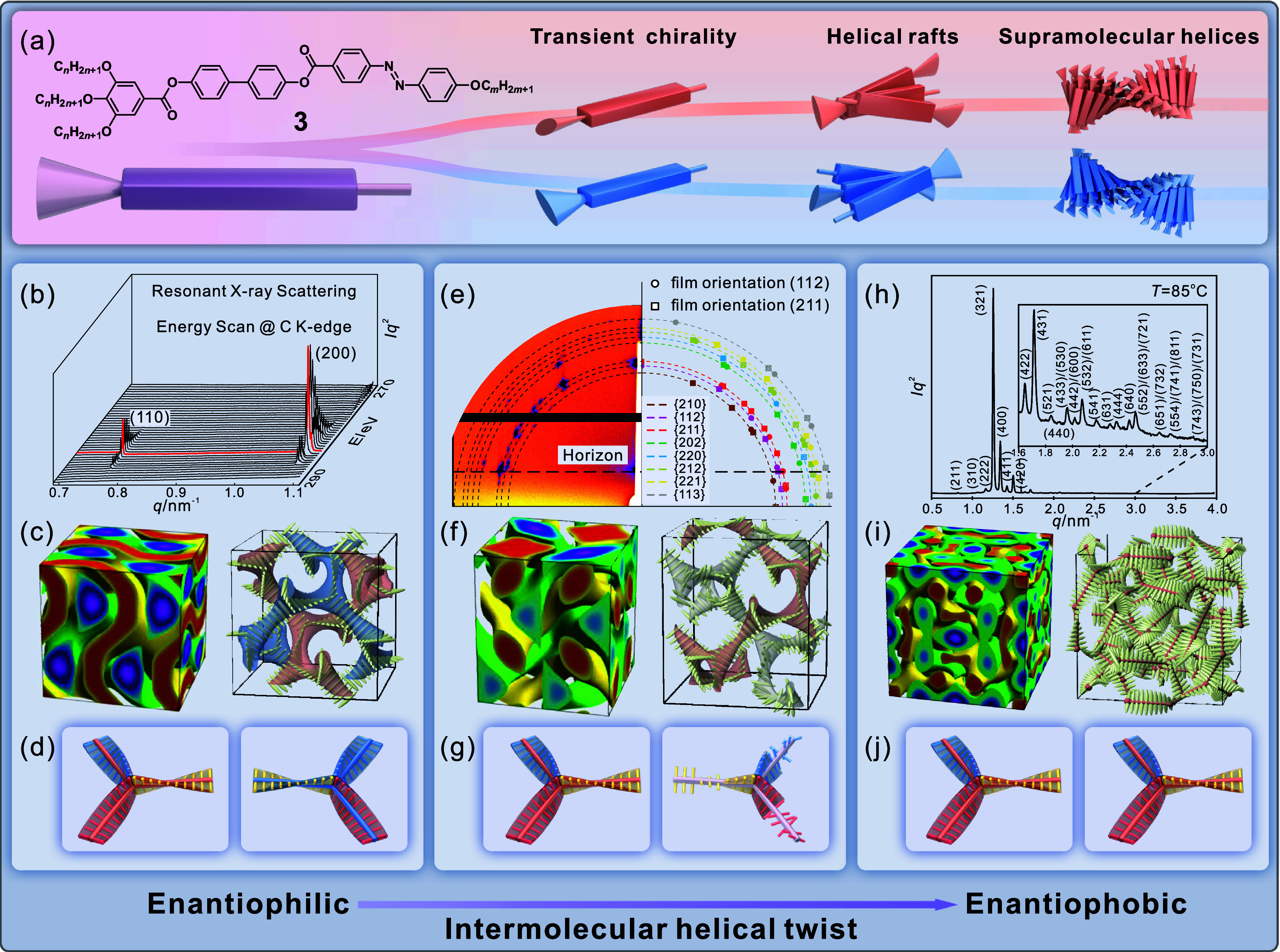
(a) The polycatenar compound **3** exhibits spontaneous helix formation which become interconnected to helical networks during self-assembly. (b) RSoXS energy scan of *Ia*3̅*d* (DG) phase at carbon K-edge. (c, f, i) Reconstructed ED maps and molecular packing models of the indicated network phases; (d, g, j) show the twist sense around the involved network junctions. (e) GISAXS pattern with simulated signal position of Tet_bi_ phase. (h) SAXS pattern of the *I*23 phase.^2,52^ [Reproduced with permission from ref 2, Copyright 2022, American Chemical Society, and ref 52, Copyright 2020, American Physical Society.]

Overall, with lowering temperature, the helical twist angle between the molecules increases and this changes the mode of helix–helix interaction from enantiophilic (→ racemate) to enantiophobic (→ conglomerate).^58,59^ The supramolecular helices in the DG phase exhibit opposite helicity in the two networks; i.e., the mode of interhelix self-assembly is enantiophilic (likely to be imposed/supported by the opposite net helicity). In a first step of the transition from enantiophilic to enantiophobic helix correlation, the chiral gyroid networks are stretched, enantiophilic helix interaction becomes less favored, and chirality synchronization along one of the networks is lost, which breaks the mirror symmetry of the double gyroid *meso*-structure in the Tet_bi_ phase. The enantiophobic self-assembly becomes dominating in the *I*23 phase, composed of three networks and containing supramolecular helices with identical sense.^44^ This provides a new mode of mirror symmetry breaking in soft self-assembled systems.^2^ Notably, the formation of the chiral *I*23 cubic network instead of the *Ia*3̅*d* (DG) *meso*-structure requires a certain range of the twist angle (*ϕ* ≈ 9°) between the molecules, leading to a transition *Ia*3̅*d* (*ϕ* ≲ 9°) – *I*23 – *Ia*3̅*d* (*ϕ* ≳ 9°) upon end-chain expansion^58–60^ or by mixing small- with large-twist *Ia*3̅*d* networks.^59,61^

Enantiophobic chirality synchronization can also be found in a tetragonal phase with the *I*4_1_22 space group, previously known as SmQ.^62^ Here, the three-way junctions in the gyroid are replaced by planar 4-way junctions, which also removes the helical twist of the network and, in such an achiral network, enantiophobic chirality synchronization of the supramolecular helices leads to uniform chirality.^62^

Uniform chirality in the double gyroid can be achieved if uniform molecular chirality, introduced by a permanently chiral stereogenic center, breaks the *meso*-structure provided by intrinsic network helicity. This leads to two different helical networks with the same helix sense, but very different intermolecular twist in the two networks; here, the DG phase itself becomes chiral (*I*4_1_32).^63^ Thus, the actual structure of the network phases is strongly influenced by the helical self-assembly of the molecules, helix orientation, and lateral interhelical interactions, which can be affected by molecular structure and temperature, i.e., by changing chain mobility, effective chain volume, and intermolecular twist angle.

In the absence of a network, in nonbranched columnar aggregates, chirality synchronization is only transmitted laterally between the columns and therefore more difficult, and this requires especially dense packing of the columns. This can either be achieved by using short alkyl chains,^64^ or by reducing temperature, as recently reported for “antiferro-chiral” (enantiophilic) helical columnar organizations.^65–67^

Ambidextrous mirror symmetry breaking events have also been observed in nematic and smectic phases of molecules with a reduced symmetry due to a pronounced bent shape,^68–71^ and just recently in polar (ferroelectric) nematic^72,73^ and lamellar phases^74,75^ of rodlike molecules.

The chirality synchronization process could inspire the development of self-sustaining and self-healing soft chiral materials for chiral polarized emission, nonlinear optics, second harmonic generation, chirality sensing,^76^ chiroptical materials,^77^ and for enantioselective synthesis and catalysis.^78^ Moreover, using achiral compounds with the capability of helical self-assembly allows the switching of the sign and magnitude of helical twist and optical activity, while these are fixed for permanently chiral compounds.

Besides mirror symmetry breaking, the networks of almost parallel aligned π-conjugated rods allow charge carrier conduction in all three spatial dimensions to minimize distortions from any potential defects.^79^ For example, with polycatenars based on [1]­benzothieno­[3,2-*b*]­benzothiophene (BTBT),^80^ the hole mobility increases from 5 × 10^–3^ to 8.2 × 10^–2^ cm^2^ V^–1^ s^–1^, at the transition from a columnar phase to the *I*23 network which can further be boosted to 0.56 cm^2^ V^–1^ s^–1^ in the crystalline cubic phase at ambient temperature, proving the network phases as efficient platform for charge carrier materials.^80^

## 3 SUPRAMOLECULAR SOFT NETWORKS BASED ON ROD–SPHERE PACKINGS

The network phases reported in the previous section belong to the so-called “bicontinuous phases”, where the helices of twisted rods form noninterrupted continuous nets in a continuum formed by the nanosegregated flexible end chains (Figure 4c). *Bicontinuous* phases, in most cases, the cubic DG phase (Figure 5a),^81^ have previously been found for the self-assembly of numerous flexible amphiphiles (lipids, detergents, block copolymers, Figure 5a).^8,82^ Notably, as no rigid rods are involved, there is no recognizable helical self-assembly in the bicontinuous cubic phases of flexible amphiphiles, as confirmed by the absence of resonant signals in RSoXS.^52^ Besides the DG with trigonal 3-way junctions, the double diamond (DD) phase^83^ with tetrahedral 4-way junctions and the double primitive (DP) phase^82,83^ with octahedral 6-way junctions are also known. All of them represent interwoven double networks with a triply periodic minimal surface (TPMS) separating them (Figure 5a).

Here, the following question arises: what happens if amphiphilic self-assembly is combined with rod packing and the flexible chains would be side-on attached to the rodlike units? Taking compounds **4** within a total of six *n*-alkyl side-chains (provided by two linear and two branched side groups) and glycerols at both ends as an example (Figure 5b),^4^ these bolapolyphiles first aggregate into bundles of parallel rods. The bundles are further stabilized and interconnected by the glycerol end-groups, forming cooperative and dynamic hydrogen bondings,^33^ which provide the junctions of the networks. This leads to a unique new type of soft network phase combining polar aggregates at the junctions with bundles of parallel π-conjugated rods as struts between them (Figure 5c–f).^3,4,84–89^ Because the rod-bundles are separated by glycerol junctions, the DG^90,91^ and DD^84^ networks are segmented and not continuous, leaving only one continuum formed by the alkyl side-chains. In this way, these *segmented network phases*, representing combinations of networks with sphere packings, are considered as *unicontinuous*. The polar spheres can also be considered as giant mesoatoms^12,92^ with distinct valences (coordination numbers = CN) and junction geometries, which are connected by the bundles of the polyaromatic rods, acting as bonds between them (Figure 5c, d).

In the series of compounds **4**, besides the DD and DG phases two noncubic phases, the orthorhombic *Fmmm* and the hexagonal *P*6_3_/*m* phase, can be observed as intermediate structures at the DD– DG transition upon side-chain volume expansion (Figure 5b-f).^4^ Taking advantage of SAXS and ED map reconstruction, we were able to reveal the molecular packing models of all four network phases. For the short-chain compound with *n* = 10, SAXS diffractograms can be indexed as a *Pn*3̅*m* space group, indicating a DD structure formed by two interwoven networks with tetrahedral four-way junctions (CN = 4, Figure 5c).^84^ Upon chain elongation, compound with *n* = 12 forms an additional mesophase with orthorhombic space group *Fmmm* containing two interwoven networks as in Figure 5e. Unlike DD with a single type of tetrahedral junctions, the *Fmmm* phase is composed by two types of junctions, deformed tetrahedral and planar four-way junctions (Figure 5e,g,h). Upon further increasing the side-chain length (*n* = 14/16), a hexagonal phase *P*6_3_/*m* forms (Figure 5f). This 3D hexagonal phase contains three interpenetrating networks with two kinds of planar three-way junctions (CN = 3), one trigonal planar and one distorted with angles unequal to 120° (Figure 5f,g,h). For compounds with longest side-chains (*n* = 18–22), the DG phase with *Ia*3̅*d* space group is confirmed (Figure 5d). In this case, the networks contain only one type of trigonal junctions. Thus, upon side-chain volume expansion, a phase sequence DD-*Fmmm- P*6_3_/*m*-DG can be observed (Figure 5g) along with a continuous transformation of the junction shape, as shown in Figure 5h.

The interjunction distance of all network phases is close to the aromatic backbone length of **4** (∼3.1 nm), suggesting all networks contain a single bundle between the junctions. There is also an acceptance region for the number of molecules in the cross section of each bundle, being 5–8 for CN = 4 and 7–8 for CN = 3 (Figure 5i). At constant CN, this number decreases with growing side-chain length to a minimum value of 5.3 molecules (Figure 5i). This number is likely to be limited by the smallest number of glycerol units being capable of forming sufficiently large hydrogen bonding networks to retain stable junctions. At least 20 glycerols are required in each sphere at the junctions. Thus, CN = 4 requires about 5 molecules and CN = 3 requires at least 7 molecules to reach this number (Figure 5i). The upper value is determined by both, minimizing the elliptical deformation of the bundle cross sections, i.e., reducing interfacial energy, and by the junction volume restriction due to limitations provided by the hexyloxy chains next to the glycerols. Apart from the DG and *P*6_3_/*m* phase, there is another hexagonal network with CN = 3 (Hex/*R*3̅*c*) formed by *p*-terphenyl based compounds as a metastable phase at the DG→honeycomb transition,^88^ at which the G-TPMS is distorted by slight variation of bond-lengths and angles.

**Figure 5:**
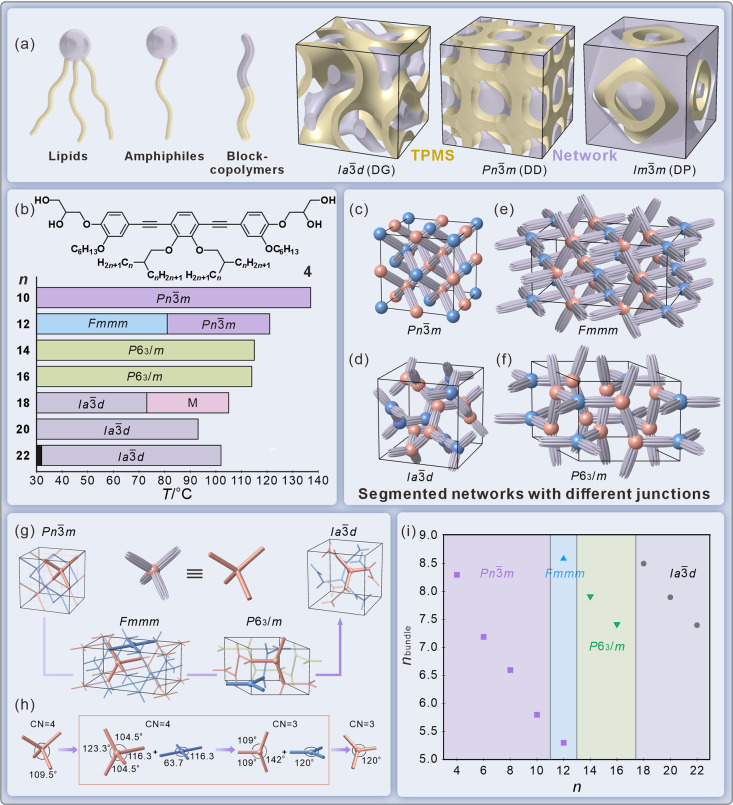
(a) Bicontinuous cubic network phases formed by flexible amphiphiles. (b) Bolapolyphiles **4** forming four different unicontinuous network phases, depending on side-chain length *n* and temperature with (c, f) molecular packing models. (g) Phase sequence and (h) junction geometry variation in the four network phases. In panels (c) and (d), a blue and orange color indicates the glycerol spheres and distinguishes the two networks, while in panels (e), (f), and (h), color is used to distinguish the junction geometries. (i) Number of molecules in the cross section of the rod bundles, depending on side-chain length and phase type.^4^ [Reproduced with permission from ref 4. Copyright 2023, American Chemical Society.]

For compounds with reduced side-chain volume, i.e. for compounds **5** with linear side chains and no chains adjacent to the glycerols, a series of three distinct network phases was observed (Figure 6).^93^ For *n* = 18, the single primitive (SP) lattice^87^ representing a cube-packing with CN = 6 is formed (Figure 6a–c). This larger CN allows maximization of the number of hydrogen bonds in the glycerol spheres whose size is no more limited by the steric restriction of peripheral substituents. As also shown in Figure 6f and g, chain elongation to *n* = 30 reduces the CN to only 4. In both cases, these long chains inhibit the interpenetration of the networks, thus leading to the noninterpenetrated, single network structures SP for *n* = 18 and single diamond (SD) for *n* = 30,^85^ which have previously not been obtained in any other bottom-up self-assembly process. Surprisingly, a double network (DG with CN = 3) is found for *n* = 22 (Figure 6d and e), being located between the two single networks. We attribute this to the capability of the glycerol aggregates at trigonal junctions to assume a prolate shape, which can enlarge the number of glycerols beyond that achievable with the almost spherical aggregates at the tetrahedral and octahedral junctions in SD and SP, respectively. As there is more space available between the nets with three-way junctions than in the meshes of the nets with larger CN, the interpenetration of two nets with CN = 3 is required to fill the larger space, and hence, a double network (DG) is formed between the two single networks with larger CNs (Figure 6c, e, g).

For the formation of networks with even larger CNs, further extension of the aromatic core length is required, as in compounds **6** (Figure 6h). Moreover, in this case the branched side-chains are positioned at opposite sides of the rod-like core. This limits the number of molecules in the cross section of the rod-bundles, as the back-to-back packing of the cores becomes difficult, thus limiting the possible strut cross-section. Here, further increasing CN to 8 is required to achieve sufficiently large glycerol aggregates, and assembly into a body centered cubic (BCC) phase with the Im3̅*m* space group was found. In this case ED reconstruction and scattering signal simulation indicate a BCC packing of spheres interconnected by the rod-bundles along the cube diagonals (Figure 6i),^86^ while a sphere packing on a simple cubic lattice (SP) is observed for **5** with significantly shorter π-conjugated rods and *n* = 18 (Figure 6a-c).^87^ For compound **6F**, the formation of the Im3̅*m* network is controlled by both, side-chain volume and core fluorination, which introduces extra steric and electronic effects, hindering the formation of honeycomb phases, leading to the novel cubic network phase with *Im*3̅*m* space group, designated as I-WP (Figure 6j).^86^

**Figure 6:**
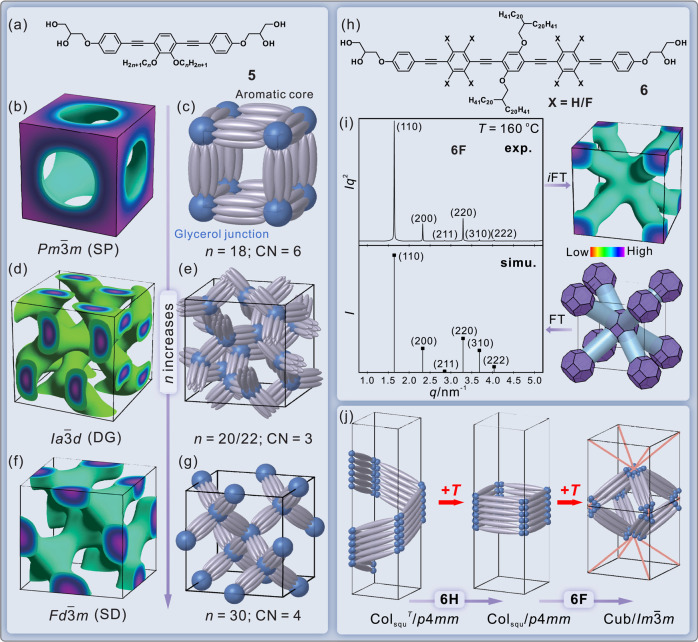
(a–g) Network phases of the bistolane based bolapolyphiles **5**, depending on the length of the side-chains (b, d, f) ED maps and (c, e, g) models of molecular organization in the networks.^93^ (h and i) Formula of compounds **6** and the polyhedral cells of the CN = 8 (I-WP) networks of **6F** (glycerol junctions are shown in purple), (j) transition from square honeycombs (*p*4*mm*) to the cubic I-WP network (*Im*3̅*m*).^86^ [Reproduced with permission from ref 86, Copyright 2020, Wiley–VCH, and ref 93, Copyright 2025, Royal Society of Chemistry.]

Similar nonfluorinated bolapolyphiles as compound **6**, but with unequal length of the branches in the side-chains, compounds **7**, form another type of cubic network (Figure 7a,b).^3,94,95^ The SAXS pattern of the cubic phase of compound **7** can be indexed to a *Pm*3̅*n* space group (Figure 7c). Upon proper phase combination, the reconstructed ED map exhibits a network with glycerol junctions of the high ED and struts of the middle ED, formed by bundles of the polyaromatic rods (Figure 7d). Two types of junctions with CN = 12 and 14 can be identified. The 14-fold junctions on the faces are larger than the 12-fold junctions at the corners and in the center of the lattice. To verify this complex network phase, a geometric model was constructed and Fourier transformed to simulate the scattering signals (Figure 7f). The striking similarity between simulation and experimental results supports the junction positions and network morphology, as shown in Figure 7f. In this network, the number of molecules in each bundle can be determined to be about 5 molecules, leading to 60 and 70 glycerols in the spheroids at the 12- and 14-fold junctions, respectively. In this network, the π-conjugated rods with a backbone length of ∼4.4 nm form nonregular tetrahedra with three different interjunction distances ranging between 4.3 and 5.3 nm. Deformation of the glycerol aggregates allows the adjustment of junction distances. The junctions of the network are sited at Wyckoff positions 2a and 6c of the *Pm*3̅*n* space group. These positions can be found in the classic Frank-Kasper A15 phase with the same *Pm*3̅*n* space group (Figure 7g). All previously known A15 phases represent packings of two types of spheres, two in the middle and at the vertices of the unit cell, and four as pairs on each face (Figure 7g).^13,96,97^ The soft spheres can be deformed to Voronoi polyhedra (dodecahedra and tetradecahedra) with a minimized interfacial area between them (Figure 7h). As a dual to the Voronoi tessellation (VT) with the *Pm*3̅*n* space group, there exists the Delaunay triangulation (DT) which fills the space with densely packed tetrahedra (Figure 7i). The DT generated tetrahedra can be obtained by connecting the adjacent points which share the common planes of the Voronoi cells as in Figure 7h,i, and this DT interconnects the spheres to a network of tetrahedra. Thus, VT and DT are combined in the A15 phase, representing a tetrahedral network of rods with spheres at the junctions.

**Figure 7:**
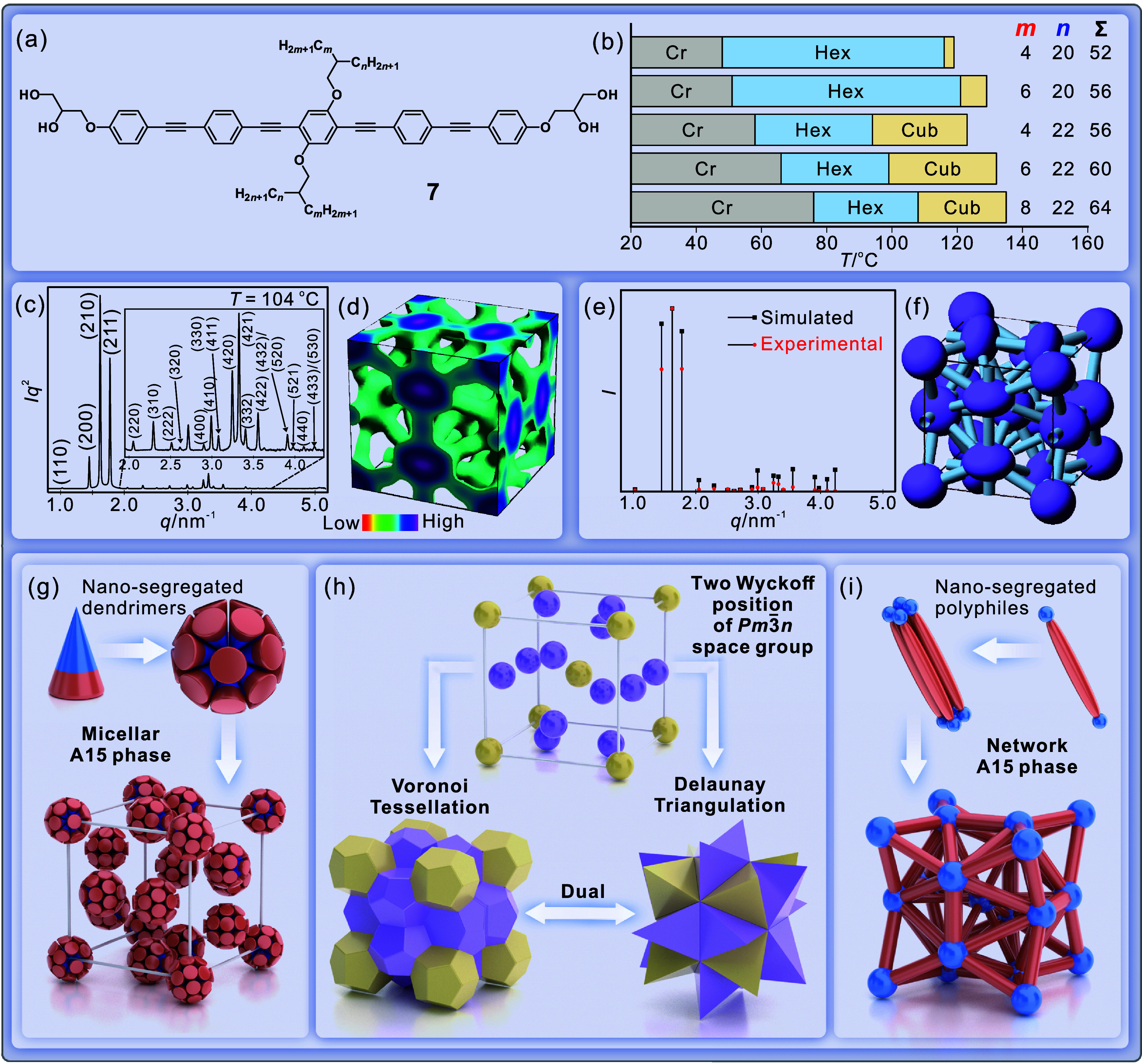
(a, b) Compound **7** forming the *Pm*3̅*n* (A15) phase and bar diagram showing the dependence of the temperature range of the *Pm*3̅*n* network depending on the side-chain structure; Hex is a hexagonal LC phase with triangular honeycomb. (c) Experimental SAXS diffractogram and (d) reconstructed ED map. (e, f) Model of the network phase and simulated SAXS signals (g–i) Duality between Voronoi tessellation and Delaunay triangulation and their representative assembly structures.^3^ Glycerol spheres are shown in purple (f) or blue (i), while in (h) the purple spheres indicate 14-way and the yellow spheres 12-way junctions. [Reproduced with permission from ref 3 Copyright 2022, Wiley–VCH.]

Figure 8 summarizes the major network phases with cubic and noncubic symmetry discovered so far.^3,4,84–90,94,95^ The actually observed network type and its CN are mainly determined by the rod length (*L*_mol_) with respect to the side-chains volume (*V*_R_). With decreasing *V*_R_/*L*_mol_ ratio and increasing CN, there are three groups of networks, double/triple networks (DG, *P*6_3_/*m*, *Fmmm*, DD), single networks (SD) and single networks with polyhedral cells (cubes in SP, octahedra in I-WP and tetrahedra in A15). Short rods such as biphenyl and *p*-terphenyl prefer low CN networks such as DG (CN = 3), DD and SD (CN = 4). The bistolane core allows the formation of additional phases like *P*6_3_/*m* (CN = 3), *Fmmm* (CN = 4) and SP with CN = 6 (Figure 8a–f), while the high-CN networks I-WP (CN = 8) and A15 (CN = 12, 14, Figure 8g, h) require even longer oligo­(phenylene ethynylene) rods with five benzene rings. At CN = 4, there appears to be the transition from double to single networks, and only for this CN both types were observed (Figure 8d, e).

In the double networks with low-CN (DG, DD) the minimization of the area of the TPMS between the two networks is the determining factor (Figure 8a, d), while with growing CN optimizing the sphere packing and adjusting the volume in the polyhedra formed by the rods and specific effects of geometric frustration become more important.

Overall, these new segmented network structures merge two different types of cubic phases—the bicontinuous and the micellar^98^—into a new mode of self-assembly unifying both in a single superstructure. Likewise, they bridge sphere packings, as found for metals and alloys, nanoparticle assemblies and colloid packings, with the reticular chemistry of organic and metal–organic frameworks (MOFs and COFs).^99–103^ Moreover, the glycerol spheres can be regarded as giant mesoatoms, with the polyaromatic rods forming the bonds between them. Being LCs, they in addition combine long-range order with mobility and molecular dynamics, leading to self-healing, adaptability, and responsive behavior. This soft assembly also allows the generation and alignment of large-scale uniform network structures with tunable pore size and geometry which after fixation and functionalization could provide materials for sensing, storage, and catalytic applications,^104^ while the single network structures are of special interest for photonic applications.^82^

**Figure 8:**
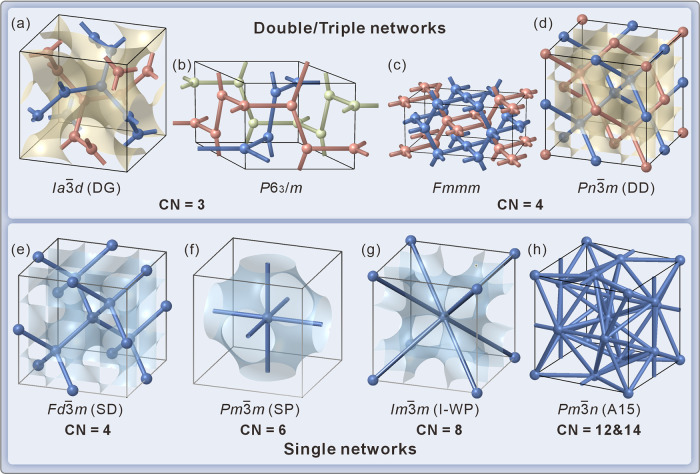
Network phases formed by bolapolyphiles. The TPMS between networks (a–d), is shown in light yellow while those in single networks (e–h) are shown in light blue.^3,4,84–90,94,95^

## 4 CONCLUSIONS AND PERSPECTIVES

In this Account, we reviewed some representative work on the structural complexity of liquid crystal self-assembly, focused on network formation, and we successfully extended the frontiers of programmable self-assembly from the domain of solid-state materials into the realm of dynamic soft matter and fluids.

The investigated systems represent π-conjugated rods with strategically positioned flexible units at the ends (polycatenars) or different groups at the ends and sides (bolapolyphiles), which determine the alignment of the rods with respect to the network direction. Tangential alignment leads to helical networks, while longitudinal alignment provides segmented networks combining rod with sphere packing.

It is shown that tangential alignment can lead to spontaneous helical self-assembly in liquids and LCs, allowing mirror symmetry breaking by cooperative chirality synchronization, representing a newly emerging field of stereochemistry with outreach into the understanding of development of uniform (bio)­chirality, and for numerous chirality discriminating chemical, chiroptical, and actuator applications.^43,50,70,105–108^

By longitudinal alignment of polyphilic molecules, new soft network structures integrating spheres into networks were developed (Figure 8) which, for example, provide access to self-assembled single networks and tetrahedral rod-packings, representing the brand-new Frank–Kasper networks. Many more of these complex modes of rod–sphere packing could be expected for the future, including those forming liquid quasicrystalline networks. This work provides the basis for soft reticular chemistry, complementing related solid-state MOFs and COFs and hydrogen-bonded frameworks.^109,110^

## Notes

Dedicated to Professor Yu Fang on the occasion of his 70th birthday.
